# Re-evaluation of FDA-approved antibiotics with increased diagnostic accuracy for assessment of antimicrobial resistance

**DOI:** 10.1016/j.xcrm.2023.101023

**Published:** 2023-04-27

**Authors:** Douglas M. Heithoff, Lucien Barnes V, Scott P. Mahan, Jeffrey C. Fried, Lynn N. Fitzgibbons, John K. House, Michael J. Mahan

**Affiliations:** 1 Department of Molecular, Cellular, and Developmental Biology, University of California, Santa Barbara, Santa Barbara, CA 93106, USA; 2 Institute for Collaborative Biotechnologies, University of California, Santa Barbara, Santa Barbara, CA 93106, USA; 3 Department of Medical Microbiology and Immunology, School of Medicine, University of California, Davis, Davis, CA 95616, USA; 4 Department of Medical Education, Santa Barbara Cottage Hospital, Santa Barbara, CA 93105, USA; 5 Department of Pulmonary and Critical Care Medicine, Santa Barbara Cottage Hospital, Santa Barbara, CA 93105, USA; 6 Division of Infectious Diseases, Santa Barbara Cottage Hospital, Santa Barbara, CA 93105, USA; 7 Faculty of Science, School of Veterinary Science, The University of Sydney, Camden, NSW 2570, Australia

**Keywords:** antimicrobial susceptibility testing, AST, minimum inhibitory concentration, MIC, antimicrobial resistance, AMR, multidrug-resistant pathogens

## Abstract

Accurate assessment of antibiotic susceptibility is critical for treatment of antimicrobial resistant (AMR) infections. Here, we examine whether antimicrobial susceptibility testing in media more physiologically representative of *in vivo* conditions improves prediction of clinical outcome relative to standard bacteriologic medium. This analysis reveals that ∼15% of minimum inhibitory concentration (MIC) values obtained in physiologic media predicted a change in susceptibility that crossed a clinical breakpoint used to categorize patient isolates as susceptible or resistant. The activities of antibiotics having discrepant results in different media were evaluated in murine sepsis models. Testing in cell culture medium improves the accuracy by which MIC assays predict *in vivo* efficacy. This analysis identifies several antibiotics for treatment of AMR infections that standard testing failed to identify and those that are ineffective despite indicated use by standard testing. Methods with increased diagnostic accuracy mitigate the AMR crisis via utilizing existing agents and optimizing drug discovery.

## Highlights

• Antibiotic testing in cell culture medium improves prediction of clinical outcome
• Antibiotics rejected by standard testing cure drug-resistant infections
• Ineffective antibiotics are identified despite indicated use by standard testing

## Graphical abstract

**Figure:**
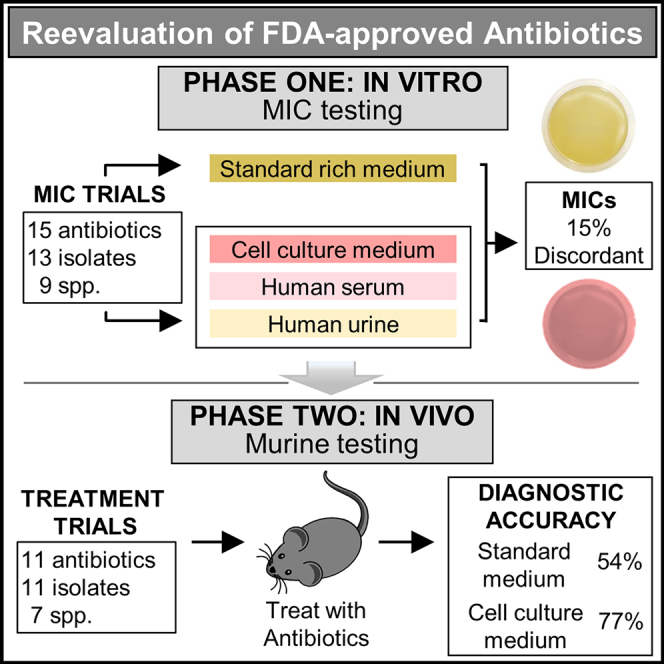

## Teaser

Heithoff et al. observe that antibiotic testing in cell culture medium improves the accuracy by which laboratory testing predicts clinical outcomes in mice. Test methods with increased diagnostic accuracy will address the antimicrobial resistance crisis by improving the way antibiotics are developed, tested, and prescribed.

## Introduction

The World Health Organization (WHO) identified antimicrobial resistance as a major threat to global health, food security, and economic stability.^1^^,^^2^ Despite the scale and urgency, few promising drug candidates are currently in the clinical pipeline due to the high costs of drug development and risk that a newly approved antibiotic becomes ineffective due to bacterial resistance or is earmarked for use as a drug of last resort.^3^^,^^4^^,^^5^ Additional factors include reduced incentives for pharmaceutical research and development for diseases that require relatively short courses of treatment (infectious diseases) relative to blockbuster drugs for pervasive diseases (cancer, cardiovascular diseases, hyperlipidemia, and immune disorders).^6^^,^^7^

The healthcare industry paradigm for the evaluation of antibiotic efficacy is based on *in vitro* assays that do not consider host-pathogen interactions that can have a marked impact on drug potency.^8^ The principal *in vitro* assay for antibiotic assessment, developed in the 1940s, uses a nutrient-rich bacteriologic medium, Mueller-Hinton broth (MHB).^9^ This assay has been used globally for antimicrobial susceptibility testing (AST) to determine the minimum inhibitory concentration (MIC), the standard measurement of antibiotic activity. MICs determine the clinical breakpoint, the concentration of antibiotic used to indicate whether an infection with a given clinical isolate is likely to be treatable in a patient.^10^^,^^11^^,^^12^ Clinical breakpoints are used by clinical microbiological laboratories to define patient isolates as susceptible (S) or resistant (R) to a panel of antibiotics. Thus, the *in vitro* MHB bioassay has been the criterion standard for guiding physician treatment practices, tracking outbreaks and epidemics, and assessing chemical structures in the development of novel therapeutics for more than half a century.

Despite these successes, *in vitro* bioassays are fundamentally flawed because antibiotic potency is highly context dependent, influenced by media composition (pH, buffers, osmolarity, nutrients); pathogen factors (load, virulence, resistance genes); host factors that can act synergistically with antimicrobials (antimicrobial peptides, complement, neutrophils); and the generation of reactive metabolic byproducts after antibiotic exposure.^13^^,^^14^ Thus, AMR therapy is often reliant on clinical reasoning by physicians on a case-by-case basis with support from agencies that provide up-to-date guidance on clinical management.^15^

Significant advances have been made to increase the accuracy by which *in vitro* assays predict clinical outcome. This is evidenced by (1) evaluation of antibacterial activity using patient serum,^16^ (2) utilization of host-mimicking media to increase predictive accuracy,^17^^,^^18^^,^^19^^,^^20^^,^^21^ and (3) antibiotic synergy with cationic antimicrobial peptides^22^^,^^23^^,^^24^ and reactive metabolic byproducts,^25^ with resultant translation to front-line therapies.^14^^,^^22^^,^^26^^,^^27^^,^^28^^,^^29^^,^^30^ However, significant hurdles remain, as many of these approaches require either patient specimens, simulation of host compartments, addition of purified biologicals, or exploitation of bacterial metabolic networks.

Here, we report the development of an alternative AST protocol for widespread clinical utility based on media that are more physiologically representative of *in vivo* infection conditions (mammalian cell culture medium, pooled human donor serum, or urine) vs. standard bacteriologic MHB medium. MHB supports the growth of bacteria and is not intended to mimic any aspect of the host environment. In contrast, cell culture medium supports the growth of mammalian cells, reflecting physiological conditions more consistent with *in vivo* sites of microbial infection, and human sera or urine are often the site/route of bacterial dissemination. MICs of clinically relevant antibiotics were evaluated against ESKAPE (*Enterococcus faecium*, *S. aureus*, *K. pneumoniae*, *A. baumannii*, *P. aeruginosa*, and *Enterobacter* spp.) pathogens (that escape the biocidal action of antibiotics)^31^ in physiologic media, and diagnostic accuracy was assessed in murine models of sepsis. Using FDA-approved antibiotics, we find that AST in mammalian cell culture medium increased diagnostic accuracy, thereby providing justification for clinical utilization of existing antibiotics for the potential treatment of AMR infections.

## Results

### Study design

The overall goal of this study was to determine whether bacterial testing in physiologic media improved the accuracy by which MIC testing predicted *in vivo* efficacy vs. that seen in standard bacteriologic MHB medium. Phase 1 of the study evaluated the MICs of clinically relevant antibiotics against clinical bacterial isolates cultured in bacteriologic MHB medium,^32^ in mammalian cell culture medium (Dulbecco’s modified Eagle medium [DMEM]),^33^ and in pooled human donor sera or urine (15 antibiotics; 13 clinical isolates; 9 bacterial spp.). The following clinical bacterial isolates were used: ESKAPE pathogens^31^ as well as *E. coli*, *S. pneumoniae*, and *S. enterica* serovar Typhimurium. Notably, two strains examined in the study, methicillin-resistant *S. aureus* (MRSA; MT3302) and carbapenem-resistant *Enterobacterales* (CRE) *K. pneumoniae* (MT3325) were derived from patients with sepsis with refractory bacteremia.^34^ Ten antibiotic classes were tested: aminoglycoside (streptomycin); β-lactam (ampicillin, cefalexin, ceftriaxone, ertapenem, imipenem, piperacillin/tazobactam); cyclic lipopeptide (daptomycin); fluoroquinolone (ciprofloxacin); glycopeptide (vancomycin); macrolide (azithromycin); oxazolidinone (linezolid); polymyxin (colistin); tetracycline (tetracycline); and sulfonamide (trimethoprim-sulfamethoxazole [co-trimoxazole]).^35^ Overall, 504 antibiotic/pathogen/media combinations were examined, including 252 Gram-positive combinations (14 antibiotics × 6 bacterial isolates × 3 media) and 252 Gram-negative combinations (12 antibiotics × 7 bacterial isolates × 3 media).

Phase 2 of the study evaluated whether bacterial testing in physiologic media improved the MIC predictive accuracy of clinical outcome vs. that seen in standard MHB medium. This *in vivo* analysis examined a total of 26 antibiotic/pathogen combinations assayed individually in murine sepsis models (11 antibiotics; 11 clinical isolates; 7 bacterial spp.). A comparative statistical analysis of the predicted number of survivors (phase 1) vs. the actual number of survivors (phase 2) was performed to determine the accuracy by which MIC testing predicted *in vivo* efficacy.

### Development of a standardized AST protocol for testing in human serum and urine

Environmental sensitization to physiologic conditions during bacterial culture and drug testing can have up to a 1,000-fold effect on antibiotic susceptibility.^20^ Thus, consideration of physiologic conditions should be implemented in a standardized AST protocol for widespread clinical utility. However, this presents a formidable challenge for test media consisting of human sera or urine that can be inhibitory to the bacterial culture of some pathogens. Although most bacterial pathogens tested exhibited robust growth after overnight culture in serum or urine pooled from human donors, several pathogens formed aggregates that impaired enumeration and subculture and/or did not support growth to adequate bacterial cell densities in microtiter plates required for reliable MIC determination.^36^ We thus established a media supplementation/aggregate disruption protocol to enable comparative AST analyses in human serum or urine (Figure S1; see STAR Methods). Briefly, bacterial isolates were sensitized to 100% pooled human donor serum or urine by overnight culture, agitated to separate bacterial cell aggregates, diluted into human fluids supplemented with 30% (v/v) Luria-Bertani broth (LB) to supply limiting nutrients,^37^ and subjected to MIC testing performed in the supplemented human fluids using microtiter plates. This procedure allowed the sensitization of bacteria in human fluids and adequate bacterial cell densities for reliable MIC determination for all pathogens tested.

### Comparative AST analysis in physiologic media vs. bacteriologic medium

**Figure 1:**
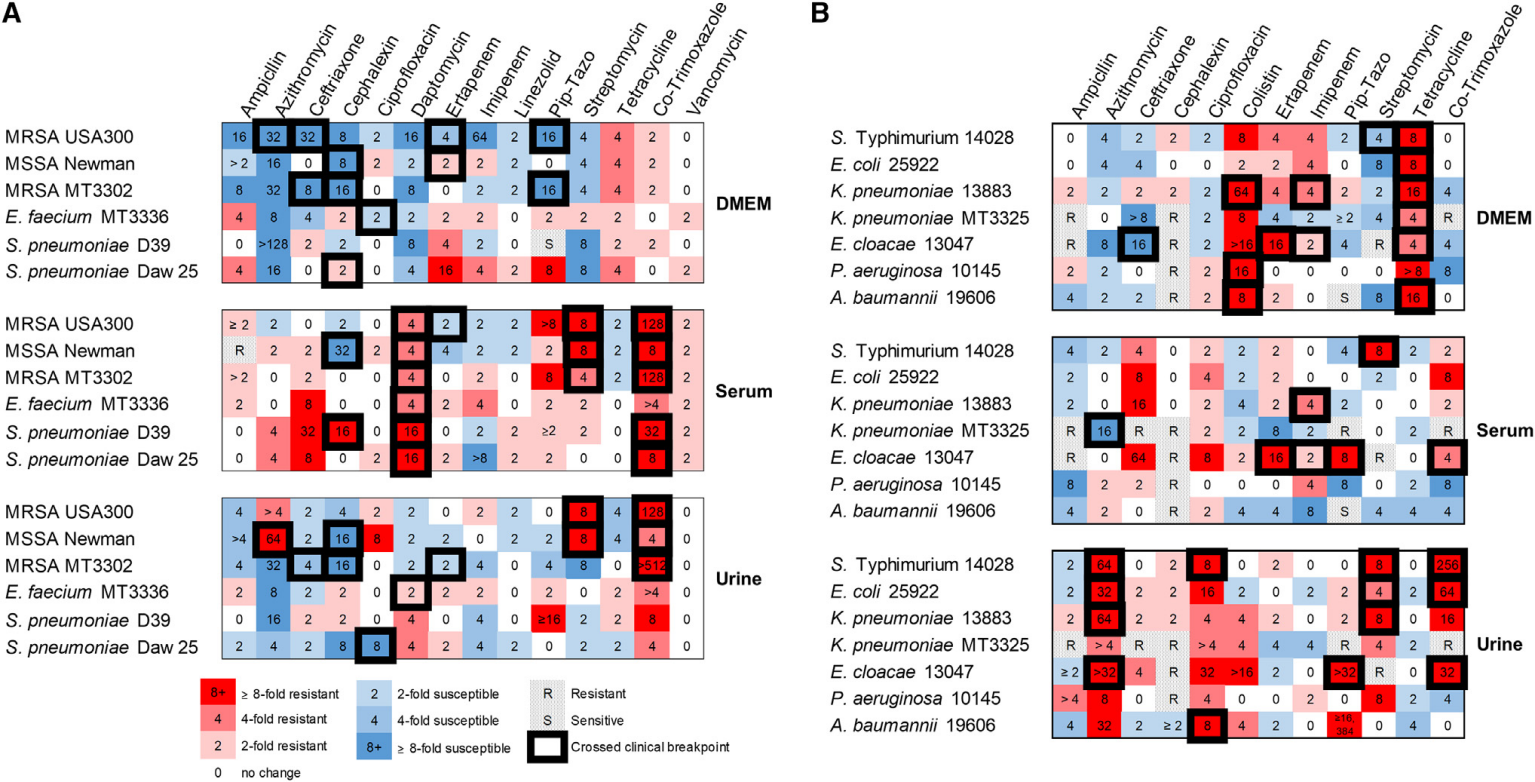
Comparative analysis of AST in cell culture medium, human sera, and urine MICs and susceptibility designations of (A) Gram-positive and (B) Gram-negative organisms were determined by broth microdilution^10^^,^^11^^,^^12^ in standard bacteriologic MHB medium, in mammalian cell culture medium (DMEM), and in pooled human donor sera or urine (15 antibiotics; 13 clinical isolates; 9 bacterial spp.; Tables S1 and S2). Values represent fold change in MICs when derived in either DMEM, human serum, or urine relative to standard MHB medium (test/standard condition). Increased susceptibility is depicted in blue; increased resistance is depicted in red; altered susceptibility designations are outlined in black boxes. Piperacillin/tazobactam (Pip/Tazo). Stippled “S” depicts intrinsic susceptibility (<0.001 μg/mL); stippled “R” depicts intrinsic resistance (>512 μg/mL). MIC values were derived from the consensus of ≥6 independent determinations.

**Figure 2:**
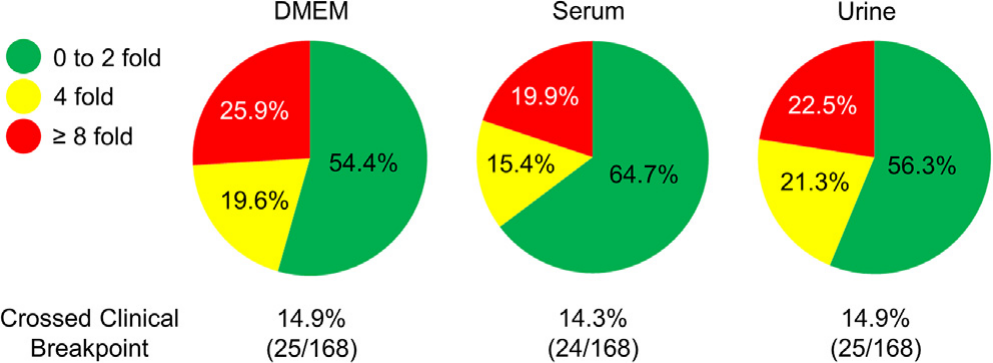
MICs and susceptibility designations derived from testing in cell culture medium, human sera, and urine Colored regions depict the fraction of pathogen-antibiotic combinations tested that exhibited a change in MIC (increased susceptibility or resistance) when derived in either mammalian cell culture medium (DMEM), pooled human donor sera, or urine relative to standard bacteriologic MHB medium; ≤2-fold (green), 4-fold (yellow), ≥8-fold (red). Percentages of pathogen-antibiotic combinations (test/standard condition) resulting in MICs that resulted in altered susceptibility designations are depicted. S, susceptible; I, intermediate; R, resistant. MICs were determined by broth microdilution.^10^^,^^11^^,^^12^

In head-to-head comparative analyses, antibiotics were evaluated for antibacterial activity against clinical isolates assayed in standard bacteriologic MHB medium, in mammalian cell culture medium (DMEM), and in pooled human donor sera or urine. Testing in physiologic media revealed that 14.7% (74/504) of the MIC values that were obtained predicted a change in susceptibility designation that crossed a clinical breakpoint (S to R; R to S) (Tables S1 and S2; Figures 1 and 2). Such altered susceptibility designations could potentially change physician decision-making provided they were supported by favorable clinical outcomes. Notably, susceptibility designations for several antibiotic/pathogen combinations derived from testing in DMEM frequently differed from those derived in MHB and in pooled human donor serum or urine. This is evidenced by the DMEM-predicted susceptibility (R to S) of (1) MRSA (USA300, MT3302) and *E. cloacae* to ceftriaxone, (2) MRSA (USA300, MT3302) to piperacillin/tazobactam, and (3) *S*. Typhimurium to streptomycin (vs. all other media predicting resistance) (Table 1), as well as DMEM-predicted resistance (S to R) of *A. baumannii*, *K. pneumoniae* and *P. aeruginosa* to colistin (vs. all other media predicting susceptibility).

**Table 1:** MIC predictive accuracy of clinical outcome in murine models of Gram-positive and Gram-negative sepsis

	MIC values (μg/mL)				Media comparisons	Mouse survivors
Pathogen/antibiotic	MHB	DMEM	Serum	Urine		
**Gram positive**						

MRSA USA300						
Ceftriaxone	256 R	8 S	256 R	128 R	DMEM vs. all media	10/10^a^
Co-trimoxazole	0.063/1.2 S	0.125/2.4 S	8/152 R	8/152 R	DMEM + MHB vs. host fluids	8/10
Ertapenem	8 R	2 S	4 I	8 R	DMEM + serum vs. MHB + urine	9/10
Pip/Tazo	64/4 R	4/4 S	>512/4 R	64/4 R	DMEM vs. all media	8/10
Streptomycin	8 S	2 S	64 R	64 R	DMEM + MHB vs. host fluids	9/10
MRSA MT3302^b^						
Ceftriaxone	64 R	8 S	128 R	16 I	DMEM vs. all media	8/10
Cephalexin	128 R	8 S	128 R	8 S	DMEM + urine vs. MHB + sera	6/10
Co-trimoxazole	0.063/1.2 S	0.125/2.4 S	8/152 R	>32/608 R	DMEM + MHB vs. host fluids	8/10
Pip/Tazo	64/4 R	4/4 S	512/4 R	16/4 R	DMEM vs. all media	8/10
MSSA Newman						
Cephalexin	32 R	4 S	1 S	2 S	MHB vs. all media	8/10
Streptomycin	8 S	2 S	64 R	64 R	DMEM + MHB vs. host fluids	10/10
*S. pneumoniae* D39						
Daptomycin	0.25 S	0.031 S	4 R	1 S	serum vs. all media	10/10
*S. pneumoniae* Daw 25						
Daptomycin	0.25 S	0.063 S	4 R	1 S	serum vs. all media	9/10

**Gram negative**						

*A. baumannii* 19606						
Ciprofloxacin	0.5 S	1 S	1 S	4 R	urine vs. all media	4/10
Colistin	0.5 S	4 R	0.125 S	2 S	DMEM vs. all media	5/10
*E. cloacae* 13047						
Azithromycin	16 S	2 S	16 S	>512 R	urine vs. all media	8/10
Ceftriaxone	4 R	0.25 S	256 R	16 R	DMEM vs. all media	7/10
*K. pneumoniae* 13883						
Azithromycin	4 S	2 S	4 S	256 R	urine vs. all media	7/10
Colistin	0.25 S	16 R	0.063 S	1 S	DMEM vs. all media	3/10
Tetracycline	1 S	16 R	1 S	1 S	DMEM vs. all media	8/10^a^
*K. pneumoniae* MT3325^b^						
Azithromycin	128 R	128 R	8 S	>512 R	serum vs. all media	0/10
Tetracycline	4 S	16 R	2 S	2 S	DMEM vs. all media	5/10
*P. aeruginosa* 10145						
Colistin	0.5 S	8 R	0.5 S	0.5 S	DMEM vs. all media	2/10
*S.* Typhimurium 14028						
Azithromycin	4 S	1 S	2 S	256 R	urine vs. all media	10/10
Co-trimoxazole	0.06/1.2 S	0.06/1.2 S	0.13/2.4 S	16/304 R	urine vs. all media	8/10
Streptomycin	16 I	4 S	128 R	128 R	DMEM vs. all media	9/10

**Figure 3:**
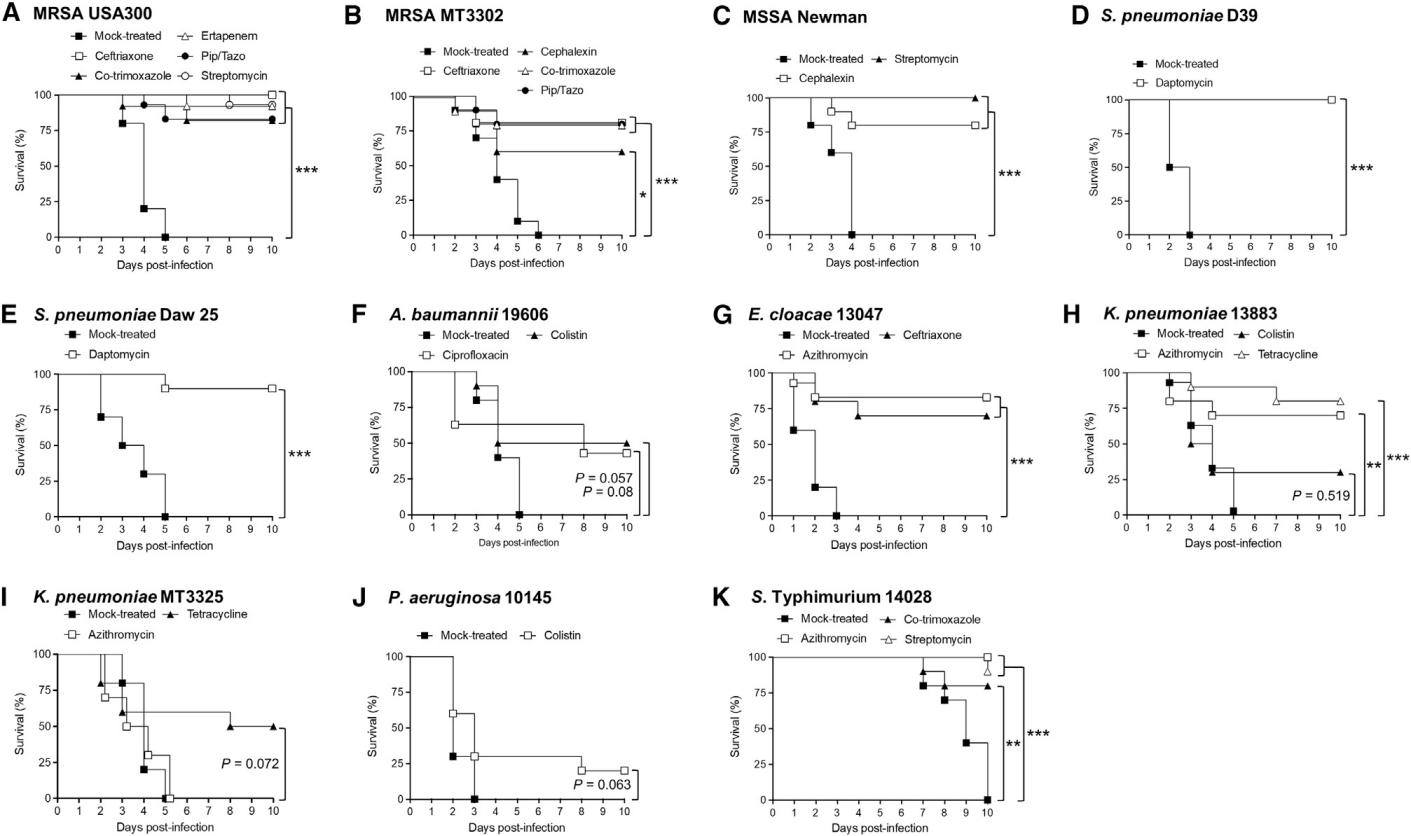
Assessment of MIC predictive accuracy in murine models of Gram-positive and Gram-negative sepsis Activities of antibiotics that had discrepant results in physiologic media (mammalian cell culture medium [DMEM], pooled human donor sera, or urine) vs. that seen in standard MHB medium were evaluated for MIC predictive accuracy of clinical outcome in murine models of sepsis (11 antibiotics; 11 clinical isolates; 7 bacterial spp.) (Table 1; see STAR Methods). Gram-positive: (A) MRSA USA300 (2 × 10^8^ CFU), (B) MRSA MT3302 (2 × 10^8^ CFU), and (C) MSSA Newman (5 × 10^8^ CFU) were administered intravenously (i.v.) to mice via retro-orbital injection, and (D) *S. pneumoniae* D39 (2 × 10^4^ CFU) and (E) *S. pneumoniae* Daw 25 (2 × 10^8^ CFU) were administered intraperitoneally (i.p.) to mice. Gram-negative: (F) *A. baumannii* ATCC 19606 (4 × 10^8^ CFU), (G) *E. cloacae* ATCC 13047 (4 × 10^8^ CFU), (H) *K. pneumoniae* ATCC 13883 (2 × 10^8^ CFU), (I) *K. pneumoniae* MT3325 (2 × 10^8^ CFU), and (J) *P. aeruginosa* ATCC 10145 (2 × 10^8^ CFU) were administered i.v. to mice by retro-orbital injection. (K) *S*. Typhimurium 14028 (10^7^ CFU) was administered to mice via gastric intubation. Survival was scored up to day 10 and compared with infected, mock-treated animals (n = 10). Pip/Tazo; ∗p < 0.05; ∗∗p < 0.01; ∗∗∗p < 0.001.

MICs and susceptibility designations were determined by broth microdilution^10^^,^^11^^,^^12^ as detailed in Tables S1 and S2. MIC values were derived from the consensus of ≥6 independent determinations. MHB and DMEM assays: unless otherwise specified, bacterial culture and testing in MHB or DMEM were performed in unsupplemented medium. Sera and urine assays: bacteria were cultured overnight in 100% pooled human donor sera or urine, agitated to separate bacterial cell aggregates, diluted into human fluids supplemented with 30% LB, and subjected to MIC testing performed in supplemented human fluids in microtiter plates (see STAR Methods) (n ≥ 6). Virulence assays: discordant MICs derived from antibiotic susceptibility testing in MHB, DMEM, human sera, and urine were examined for diagnostic accuracy following individual assay in murine sepsis models (n = 10) (see Figure 3; STAR Methods). Pip/Tazo, piperacillin/tazobactam; S, susceptible; I, intermediate; R, resistant.

a Survivorship in Ersoy et al.^19^

b Bacterial isolate derived from a patient refractory to antibiotic therapy.

### Assessment of MIC predictive accuracy of clinical outcome in murine models of Gram-positive and Gram-negative sepsis

The activities of antibiotics that had discrepant results in physiologic media were evaluated for MIC predictive accuracy of clinical outcome vs. that seen in standard MHB medium. This *in vivo* analysis constituted the individual assay of 26 antibiotic/pathogen combinations in murine models of sepsis (11 antibiotics; 11 clinical isolates; 7 bacterial spp.) (Figure 3; Table 1). The dose/route of infection and sepsis disease progression was based on established animal models of sepsis (see STAR Methods).^38^^,^^39^ All mice in the mock-treated groups died, providing an indication of expected mortality in the absence of effective treatment. Briefly, pairwise comparisons of test accuracy were performed between the media across all pathogen and antimicrobial combinations and between MHB and DMEM for each antibiotic and pathogen (see STAR Methods). Diagnostic accuracy was calculated as the number of animals that were predicted to survive and did survive combined with the number of animals that were predicted to succumb and did succumb divided by the total numbers of animals. Statistical analyses returning a p value of <0.05 were considered significant. Diagnostic accuracy of discordant MICs that crossed a clinical breakpoint increased from 54% in MHB to 77% in DMEM (p = 0.014), but accuracy decreased to 34% in pooled human donor sera or urine (p = 0.006)*.* Increased diagnostic accuracy in cell culture medium was a reflection of improved prediction of antibiotic treatment success from 61% in MHB to 87.7% in DMEM (p = 0.026) and a trend for improved prediction of treatment failure from 37% in MHB to 50.7% in DMEM (p = 0.37).

Increased diagnostic accuracy in DMEM was demonstrated in several animal models of infection vs. that seen in MHB and in pooled human donor serum or urine. This is evidenced by the DMEM-predicted treatment success (R to S) of (1) MRSA (USA300, MT3302) and *E. cloacae* with ceftriaxone (Figures 3A, 3B, and 3G), (2) MRSA (USA300, MT3302) with piperacillin/tazobactam (Figures 3A and 3B), and (3) *S*. Typhimurium with streptomycin (Figure 3K) (vs. all other media predicting resistance), as well as DMEM-predicted treatment failures (S to R) of *A. baumannii*, *K. pneumoniae* (13883), and *P. aeruginosa* with colistin (Figures 3F, 3H, and 3J) (vs. all other media predicting susceptibility). An exception is the treatment success (but DMEM-predicted failure) of *K. pneumoniae* (13883) with tetracycline (Figure 3H).

Notably, diagnostic accuracy of MHB increased to 84% when test results were in agreement with DMEM (p < 0.001) but fell to 29% when in disagreement (p < 0.001); diagnostic accuracy of DMEM remained unchanged when in disagreement with MHB (71.4%; p = 0.4). Improved test accuracy achieved when the two media were in agreement was evidenced by the treatment success (S and S) of (1) MRSA (USA300; MT3302) and *S*. Typhimurium with co-trimoxazole (Figures 3A, 3B, and 3K); (2) MRSA (USA300) and MSSA with streptomycin (Figures 3A and 3C); (3) *S. pneumoniae* (D39; Daw25) with daptomycin (Figures 3D and 3E); and (4) *E. cloacae*, *K. pneumoniae* (13883), and *S*. Typhimurium with azithromycin (Figures 3G, 3H, and 3K), as well as treatment failure (R and R) of *K. pneumoniae* (MT3325) with azithromycin (Figure 3I). Taken together, these findings suggest that testing in DMEM cell culture medium, either alone or in combination with standard bacteriologic MHB medium, provides an approach to identify presently available antibiotics for the potential treatment of AMR infections.

## Discussion

Our results indicate that re-evaluation of existing FDA-approved antibiotics may be an important augmentation to the development of new drugs to combat antimicrobial resistance. In head-to-head comparisons of physiologic media (DMEM, sera, urine) vs. standard bacteriologic MHB medium, ∼15% of the MIC values obtained in physiologic media predicted a change in susceptibility that crossed a clinical breakpoint, the concentration of antibiotic used to define whether an infection with a given clinical isolate is likely to be treatable in a patient. Diagnostic accuracy of these discordant MICs increased when the testing was carried out in DMEM cell culture medium and assayed in murine models of Gram-positive and Gram-negative sepsis. The test advancement was a reflection of both improved prediction of antibiotic treatment success and a trend for improved prediction of antibiotic treatment failure. This led to the identification of potentially effective FDA-approved antibiotics for the treatment of AMR infections that standard testing failed to identify and also excluded those that were ineffective despite indicated use by standard testing. Additionally, diagnostic accuracy of bacteriologic medium increased when test results were in agreement with cell culture medium results but dropped when in disagreement. Thus, test agreement between the two culture test conditions may increase confidence for clinical decision-making, while contrary predictions may favor an adjunctive therapy to primary treatment. Clinical implementation of test methods with improved diagnostic accuracy provides a platform to expand the therapeutic armamentarium, improve clinical management and antibiotic stewardship, and facilitate the discovery of novel compounds with improved pharmacological properties.

This study provides a potential solution for addressing discrepant results between antibiotics indicated by standard AST and actual clinical outcomes. Indeed, the limitations of standard AST methods for predicting clinical efficacy are being increasingly recognized, as certain antibiotics dismissed by standard testing are effective at treating AMR infections. This is evidenced by β-lactams as adjunctive therapy for refractory bacteremia caused by MRSA and vancomycin-resistant *Enterococcus*,^22^^,^^26^ azithromycin monotherapy for multidrug-resistant *P. aeruginosa*,^28^ and azithromycin/piperacillin-tazobactam combination therapy for CRE *Achromobacter xylosoxidans*.^40^ Further, despite limited azithromycin breakpoint designations for Enterobacterales (*S.* Typhi and *Shigella* spp.),^41^^,^^42^ azithromycin has been used clinically for diarrheagenic *E. coli*, *Shigella* spp., *Salmonella* spp., and *Campylobacter* spp.^43^^,^^44^ This has led to proposed azithromycin breakpoints for *diarrheagenic E. coli*,^45^ and azithromycin is being considered as a standard therapy for specific Enterobacterales infections.^45^^,^^46^^,^^47^

The mechanism by which DMEM cell culture medium improved the accuracy by which MIC testing predicted *in vivo* efficacy appears to rely on the presence of physiologic levels of sodium bicarbonate, an anionic buffer that plays a role in the maintenance of blood and tissue pH.^48^ However, the role of bicarbonate in antibiotic susceptibility was not simply a reflection of pH stabilization^49^ because removal of NaHCO_3_ from exogenously buffered DMEM cell culture medium resulted in MICs similar to MHB in many species,^19^ and, reciprocally, the addition of NaHCO_3_ to exogenously buffered MHB resulted in MICs similar to DMEM cell culture medium. Rather, bicarbonate is a pleiotropic ionic factor that (1) stimulates global changes in bacterial gene expression with resultant changes in bacterial membrane permeability that impact susceptibility to cationic peptides,^49^ (2) affects bacterial virulence gene expression and resultant susceptibility to β-lactam antibiotics,^50^ and (3) contributes to the dissipation of the bacterial proton motive force (PMF) required for activity or import/export of various classes of antibiotics and several immune components (defensins, cathelicidins, bile salts).^51^ Additionally, such changes in antibiotic susceptibility might also have an indirect effect on bactericidal action via stimulating host cytokine responses important for bacterial clearance. For example, bicarbonate-mediated changes in bacterial membrane permeability can increase β-lactam levels. β-Lactams can increase the expression of α-toxin in *S. aureus*,^52^ which in turn can prompt an immunostimulatory interleukin-1-β (IL-1-β) response^53^ with resultant enhanced host recognition/bacterial clearance for the successful treatment of *S. aureus* bacteremia.^54^^,^^55^

Testing in each of the three physiologic media examined (DMEM, human sera, or urine) resulted in a similar fraction of MICs (∼15%) that predicted a change in clinical breakpoint classification. The increased diagnostic accuracy of DMEM relative to the other physiologic media tested is not driven solely by the presence of bicarbonate, as it is also present in human sera and urine (DMEM, 44 mM; sera, ∼25 mM; urine, ∼2.5 mM).^33^^,^^48^^,^^56^^,^^57^ It may, however, reflect that DMEM supports the growth of mammalian cells, emulating physiological conditions more consistent with *in vivo* sites of microbial infection. An alternate possibility is that results concerning the predictive power of human serum or urine (vs. DMEM or MHB) do not apply to mice but may apply to the human condition because of inherent milieu differences between the two species that impact drug potency. Notably, results of animal models of systemic infection may not be readily translated to other modes of infection, including respiratory, skin, urinary tract infections (UTIs), or even some cases of bacteremia (given the route of infection of the models used), and thus individual physiologic media might be more predictive for their corresponding site of infection. Therefore, conclusions concerning the predictive power of serum or urine (vs. DMEM or MHB) require further investigation using additional models of infection (e.g., respiratory, skin, UTIs).

AST in physiologic media may impact the means by which antibiotics are tested, developed, and prescribed and offers a number of advantages over conventional methods. Foremost is improved diagnostic accuracy. Additional advantages include growth support of most pathogens observed in clinical practice and ease of adoption to existing protocols/instrumentation—making the methodological transition of culture conditions simple, scalable, and affordable. Refined AST methods have potential benefit to both empiric antimicrobial therapy (prior to the receipt of blood culture and AST results) and definitive antimicrobial therapy (subsequent to blood culture and AST results),^58^ which may ultimately improve clinical management and patient outcome. Testing in mammalian cell physiologic media exemplified by DMEM provides a platform for evaluation both of FDA-approved antibiotics and other compounds under development, potentially leading to significant cost and life savings.

### Limitations of the study

*In vitro* assays are subject to inherent limitations since they fail to recapitulate the full spectrum of interactions of antibiotics between the intact animal host and pathogen that are highly heterogeneous in time and space. Antibiotic concentrations can be modulated by absorption, distribution, metabolism, and excretion and further influenced by the dynamic nature of the infective process (nutrient availability, innate immune synergy, reactive metabolic product synergy).^13^^,^^14^^,^^59^ Although improved diagnostic accuracy in DMEM was observed across a diversity of bacterial species and antimicrobials, these findings cannot be conclusive or generalized for MIC determination of individual bacterial species without increasing the number of clinical isolates tested to ensure sufficient clinical representation. Additionally, clinical outcomes derived from systemic infection may not apply to localized infections (respiratory, skin, UTIs), and thus testing in physiologic media more representative of the corresponding site of infection might increase the accuracy by which MIC assays predict *in vivo* efficacy. Further, human clinical efficacy and toxicity studies will need to be conducted to assure that these findings are applicable to patients with various infections and sepsis.

## STAR★Methods

### Key resources table

**Table:** 

REAGENT or RESOURCE	SOURCE	IDENTIFIER
**Bacterial and virus strains**		

*Acinetobacter baumannii*	ATCC 19606	2208
*Enterobacter cloacae*	ATCC 13047	CDC 442-68
*Klebsiella pneumoniae*	ATCC 13883	NCTC 9633
*Klebsiella pneumoniae*	Heithoff et al.^34^	CRE MT3325
*Pseudomonas aeruginosa*	ATCC 10145	(Schroeter) Migula
*Salmonella enterica* serovar Typhimurium	ATCC 14028	CDC 6516-60
*Staphylococcus aureus*, methicillin-resistant	Diekema et al.^60^	CA-MRSA USA300
*Staphylococcus aureus*, methicillin-resistant	Heithoff et al.^34^	MRSA MT3302
*Staphylococcus aureus*, methicillin-sensitive	Yang et al.^38^	MSSA Newman
*Streptococcus pneumoniae*	Lanie et al.^61^	D39 (ser. 2)
*Streptococcus pneumoniae*	Carter et al.^62^	Daw 25 (ser. 35C)

**Biological samples**		

Human donor sera	Millipore Sigma	Cat #S1-LITER
Human donor urine	Innovative Research	Cat # 50-203-6075

**Chemicals, peptides, and recombinant proteins**		

Columbia CNA agar with 5% sheep blood	Becton Dickinson	Cat #221352
Dulbecco’s Modified Eagle Medium (DMEM, High Glucose)	Life Technologies	Cat #11965-092
Mueller-Hinton Broth (MHB)	Becton Dickinson	Cat # 275730
Todd-Hewitt Broth (THB)	Becton Dickinson	Cat # 249240
Tryptic Soy Broth (TSB)	Becton Dickinson	Cat # 211825

**Experimental models: Organisms/strains**		

C57BL/6J mice	The Jackson Laboratory	N/A

**Software and algorithms**		

GraphPad Prism (v9.2.0)	GraphPad Software	https://www.graphpad.com/updates/prism-900-release-notes
R Statistical Software (v4.2.0)	R Core Team	https://cran.r-project.org/bin/windows/base/old/4.2.0/
epiR R package (v2.0.52)	Tools for the Analysis of Epidemiological Data	https://rdocumentation.org/packages/epiR/versions/2.0.52

### Resource availability

#### Lead contact

Further information and requests for resources and reagents should be directed to the lead contact, Michael J. Mahan (mahan@ucsb.edu).

#### Materials availability

This study did not generate new unique reagents.

### Experimental model and subject details

#### Bacterial strains and culture conditions

##### Bacterial strains

Gram-positive bacterial isolates included: methicillin-resistant *Staphylococcus aureus*, MRSA USA300^60^ and MRSA MT3302 (refractory bacteremia isolate);^34^ methicillin-sensitive *S. aureus* (MSSA) Newman;^38^ *Enterococcus faecium* MT3336, human blood isolate; and *S. pneumoniae* D39 (ser. 2),^61^ and Daw 25 (ser. 35C).^62^ Gram-negative bacterial isolates included: *Salmonella enterica* subsp. *enterica* serovar Typhimurium ATCC 14028;^63^ *Escherichia coli* ATCC 25922;^19^ *Klebsiella pneumoniae* ATCC 13883;^64^ carbapenem-resistant *Enterobacterales* (CRE) *K. pneumoniae* MT3325 (refractory bacteremia isolate);^34^ *Enterobacter cloacae* ATCC 13047;^65^ *Pseudomonas aeruginosa* ATCC 10145;^66^ and *Acinetobacter baumannii* ATCC 19606.^67^

##### Bacteria culture conditions

Gram-positive *S. aureus* and *E. faecium* were isolated on Tryptic Soy Broth (TSB) agar incubated at 37^o^C in ambient air. *S. pneumoniae* strains were grown overnight on Columbia CNA agar with 5% sheep blood (Becton Dickinson), grown in Todd-Hewitt Broth (THB) supplemented with 2% yeast extract, and incubated at 37^o^C in a 5% CO_2_ incubator. Gram-negative bacteria were isolated on Luria-Bertani (LB) agar^37^ and incubated at 37^o^C in ambient air. Standard AST broth medium was Mueller-Hinton Broth (MHB) supplemented with CaCl_2_ and MgCl_2_ to make cation-adjusted MHB (Ca-MHB).^32^ Mammalian cell culture medium was Dulbecco's Modified Eagle Medium (DMEM, High Glucose [Life Technologies]).^33^

#### Virulence studies

The route of infection, infectious inoculum for each strain, and the observed progression of sepsis (pre-infection, pre-disease, and sepsis) was based on previously reported literature^38^^,^^39^ and is summarized below. A dose of 20 × LD_50_ ensures that virtually all animals will undergo sepsis and was the inoculum used in this study. *Gram-negative*: *S*. Typhimurium 14028 (10^7^ cfu) was grown overnight in LB, resuspended in sterile 0.2M Na_2_HPO_4_ pH 8.1 buffer, and administered to mice via gastric intubation; t=0 (pre-infection), t=5 days (pre-disease), t=8 days (sepsis). *A. baumannii* ATCC 19606 (4 × 10^8^ cfu); *E. cloacae* ATCC 13047 (4 × 10^8^ cfu); *K. pneumoniae* ATCC 13883 (2 × 10^8^ cfu); *K. pneumoniae* MT3325 (2 × 10^8^ cfu), and *P. aeruginosa* ATCC 10145 (2 × 10^8^ cfu) were grown overnight in LB medium, resuspended in sterile PBS, and administered i.v. to mice by retro-orbital injection. *A. baumannii* ATCC 19606, *K. pneumoniae* ATCC 13883, *K. pneumoniae* MT3325, t=0 (pre-infection), t=24 h (pre-disease), t=72 h (sepsis); *E. cloacae* ATCC 13047, t=0 (pre-infection), t=12 h (pre-disease), t=24 h (sepsis); *P. aeruginosa* ATCC 10145, t=0 (pre-infection), t=24 h (pre-disease), t=48 h (sepsis). *Gram-positive*: MRSA USA300 (2 × 10^8^ cfu); MRSA MT3302 (2 × 10^8^ cfu) and MSSA Newman (5 × 10^8^ cfu) were grown overnight in TSB and sub-cultured to A_600_ = 0.4, resuspended in sterile PBS and administered i.v. to mice by retro-orbital injection; t=0 (pre-infection), t=24 h (pre-disease), and t=48 h for MRSA and 96 h for MSSA (sepsis). *S. pneumoniae* D39 (2 × 10^4^ cfu) and Daw 25 (2 × 10^8^ cfu) were grown overnight in THB with 2% yeast extract and sub-cultured to A_600_ = 0.4, resuspended in sterile PBS and administered i.p. to mice, t=0 (pre-infection), t=24 h (pre-disease), and t=48 h (sepsis). Equal numbers of male and female 10- to 12-week-old littermate C57BL/6J mice were used in all virulence studies. Institutional Animal Care and Use Committee of the University of California, Santa Barbara approved all mouse research protocols undertaken herein.

### Method details

#### MIC assays

MICs were determined by broth microdilution according to the Clinical and Laboratory Standards Institute (CLSI) and European Committee on Antimicrobial Susceptibility Testing (EUCAST).^10^^,^^11^^,^^12^ MIC values were derived from the consensus of ≥ 6 independent determinations, whereby the reported value was observed in ≥ 4 of 6 determinations. In cases where 6 determinations were not sufficient to determine a consensus MIC value, additional triplicate determinations were performed until a consensus value was obtained. MHB and DMEM assays: Bacterial isolates were sensitized to MHB or DMEM by overnight culture and equivalent bacterial cfu (5 x 10^5^ cfu/mL) were used for MIC determination of all pathogens tested. *S. aureus* MIC assays were performed by direct inoculation: five to seven *S. aureus* colonies from TSB agar were used to inoculate 1 mL Ca-MHB or 1 colony was used to inoculate 0.5 mL DMEM with 5% LB. *S. pneumoniae* was grown overnight on Columbia CNA agar with 5% sheep blood, and 5 colonies were inoculated into 0.5 mL Ca-MHB supplemented with 5% lysed horse blood (Lampire Biological Laboratories), and incubated 4 h at 37^o^C in a 5% CO_2_ incubator. Sera and urine assays: Bacterial isolates were sensitized to 100% pooled human donor sera (Millipore Sigma) or urine (Innovative Research) by overnight culture (5 x 10^7^ cfu/mL to 3 x 10^9^ cfu/mL); agitated to separate bacterial cell aggregates; diluted into human fluids supplemented with 30% Luria-Bertani broth (LB) to supply limiting nutrients; and subjected to MIC testing performed in supplemented human fluids in microtiter plates (Figure S1). MICs were obtained after 20 h incubation at 37^o^C in ambient atmosphere without shaking (Ca-MHB, urine) or 5% CO2 incubator (DMEM, serum). *A. baumannii* required heat-inactivated serum with 40% v/v MHB supplementation for bacterial culture and MIC testing. Equivalent bacterial cfu (1 x 10^6^ cfu/mL) were used for MIC determination of all pathogens tested.

#### Antibiotic treatment

Infected mice were treated (or mock-treated) with the following dosing regimens beginning 2 h post-infection: azithromycin (100 mg/kg/day),^23^ ceftriaxone (50 mg/kg/day),^68^ cephalexin (50 mg/kg/day),^69^ ciprofloxacin (30 mg/kg/day),^70^ colistin (30 mg/kg/day),^71^ co-trimoxazole (75 mg/kg/day sulfamethoxazole; 15 mg/kg/day trimethoprim),^72^ daptomycin (10 mg/kg/day),^73^ ertapenem (60 mg/kg/day),^74^ piperacillin/tazobactam (200 mg/kg/day piperacillin; 25 mg/kg/day tazobactam),^75^ streptomycin (75 mg/kg/day),^72^ or tetracycline (100 mg/kg/day).^76^ All drug doses were delivered by the i.p. route once every 12 h except ertapenem, which was delivered once every 8 h. Mouse survival was assessed for 10 days post-infection.

#### Ethics statement

Human subjects approval was obtained from the Institutional Human Subjects Use Committee of the University of California, Santa Barbara and the Institutional Review Board of Santa Barbara Cottage Hospital. All animal experimentation was conducted following the National Institutes of Health guidelines for housing and care of laboratory animals and performed in accordance with Institutional regulations after pertinent review and approval by the Institutional Animal Care and Use Committee at the University of California, Santa Barbara.

### Quantification and statistical analyses

#### Statistical analysis of mouse survival

Log-rank (Mantel-Cox) test was used to compare differences in survival between groups for Kaplan-Meier survival curves; significance was determined using GraphPad Prism version 9.2.0. *P* values of less than 0.05 were considered significant (n =10/cohort).

#### Statistical analysis of predicted & actual outcome

Antimicrobial susceptibility in murine sepsis models was evaluated using R Statistical Software (v4.2.0).^77^ Statistical analyses returning a *p* value of <0.05 were considered significant. Experimental challenge experiments evaluated outcomes for multiple antimicrobials and bacterial species focusing on scenarios where there were discrepant classifications of antimicrobial susceptibility between tests performed utilizing different antimicrobial susceptibility testing media. All mice in the mock treated groups died providing an indication of expected mortality in the absence of effective treatment. Correct classification of susceptibility to an antimicrobial was anticipated to be associated with an increased proportion of mice surviving challenge. Conversely, an antimicrobial susceptibility classification of resistance was anticipated to be associated with an increased proportion of mice succumbing to challenge. The relationship between the susceptibility classification provided by each susceptibility testing method was compared by determining the proportion of animals that survived and died following challenge. Susceptibility testing method accuracy reflected the proportion of individual mouse outcomes that were consistent with the susceptibility predictions of the testing method where antimicrobial susceptibility was assigned a prediction of survival (the desired outcome should the antimicrobial be utilized in clinical practice) and antimicrobial resistance was assigned mortality (the outcome observed with mock treatment). Pairwise comparisons of test accuracy were performed between the media across all pathogen and antimicrobial combinations; further pairwise comparisons of test accuracy were performed between MHB and DMEM for each antibiotic and pathogen. EUCAST recommendations call for dose adjustments for “intermediate” susceptibility (I)^78^ and, thus for statistical analysis, test results were dichotomized to either susceptible or not (intermediate or resistant) consistent with the EUCAST recommendations for standard antimicrobial dosing. Diagnostic accuracy was calculated as the number of animals that were predicted to survive and did survive, combined with the number of animals that were predicted to succumb and did succumb, divided by the total numbers of animals. For this statistical analysis, the predicted number of survivors for susceptibility (10/10 animals) and intermediate/resistance (0/10 animals) was compared to the actual number of survivors observed. The accuracy of antimicrobial susceptibility testing methods was calculated using the epiR R package (v2.0.52).^79^ Fisher’s exact test with false discovery rate (FDR) adjustment for multiple comparisons was used to compare the accuracy of susceptibility test methods using the RVAideMemoire R package (v0.9-81-2).

## Data Availability

• All data reported in this paper will be shared by the lead contact upon request. • This study did not generate new sequencing data or code. • Any additional information required to reanalyze the data reported in this paper is available from the lead contact upon request.

## Supplemental information

Document S1. Figure S1 and Tables S1 and S2

Document S2. Article plus supplemental information
