# Supplementary material for: Re-evaluation of FDA-approved antibiotics with increased diagnostic accuracy for assessment of antimicrobial resistance
Source: Cell Rep Med. 2023 Apr 27;4(5):101023. doi: 10.1016/j.xcrm.2023.101023 (PMC10213814; doi:10.1016/j.xcrm.2023.101023)
Supplement: Document S1. Figure S1 and Tables S1 and S2 [file mmc1.pdf]

**Cell Reports Medicine, Volume *4***

## Supplemental information

# Re-evaluation of FDA-approved antibiotics with increased diagnostic accuracy for assessment of antimicrobial resistance

**Douglas M. Heithoff, Lucien Barnes V, Scott P. Mahan, Jeffrey C. Fried, Lynn N. Fitzgibbons, John K. House, and Michael J. Mahan**

## Supplementary Tables

**Table S1. Gram-positive MICs and susceptibility designations from testing in bacteriologic medium, cell culture medium, and human sera or urine.** Related to Figure 1.

***E. faecium* (MT3336)**

| | MHB | | DMEM | | Serum | | Urine | |
|---|---|---|---|---|---|---|---|---|
| Antibiotic | MIC | Interpretation | MIC | Interpretation | MIC | Interpretation | MIC | Interpretation |
| Ampicillin | 0.25 | S | 1 | S | 0.5 | S | 0.5 | S |
| Azithromycin | 0.5 | S | 0.063 | S | 0.5 | S | 0.063 | S |
| Ceftriaxone* | 8 | R | 2 | R | 64 | R | 4 | R |
| Cephalexin* | 64 | R | 128 | R | 64 | R | 128 | R |
| Ciprofloxacin | 2 | I | 1 | S | 2 | I | 2 | I |
| Daptomycin | 4 | S | 2 | S | 16 | R | 8 | R |
| Ertapenem | 8 | R | 16 | R | 16 | R | 16 | R |
| Imipenem | 0.5 | S | 1 | S | 2 | S | 1 | S |
| Linezolid | 2 | S | 2 | S | 2 | S | 2 | S |
| Piperacillin/Tazobactam | 2/4 | S | 4/4 | S | 4/4 | S | 4/4 | S |
| Streptomycin* | 32 | R | 64 | R | 64 | R | 32 | R |
| Tetracycline | 0.125 | S | 0.25 | S | 0.125 | S | 0.25 | S |
| Trimethoprim/Sulfamethoxazole* | 8/152 | R | 8/152 | R | >32/608 | R | >32/608 | R |
| Vancomycin | 0.5 | S | 1 | S | 1 | S | 0.5 | S |

**MRSA USA300**

| | MHB | | DMEM | | Serum | | Urine | |
|---|---|---|---|---|---|---|---|---|
| Antibiotic | MIC | Interpretation | MIC | Interpretation | MIC | Interpretation | MIC | Interpretation |
| Ampicillin | 512 | R | 32 | R | >512 | R | 128 | R |
| Azithromycin | 128 | R | 4 | I | 64 | R | >512 | R |
| Ceftriaxone | 256 | R | 8 | S | 256 | R | 128 | R |
| Cephalexin | 256 | R | 32 | R | 128 | R | 64 | R |
| Ciprofloxacin | 0.5 | S | 0.25 | S | 0.5 | S | 1 | S |
| Daptomycin | 1 | S | 0.063 | S | 4 | R | 0.5 | S |
| Ertapenem | 8 | R | 2 | S | 4 | I | 8 | R |
| Imipenem | 2 | S | 0.031 | S | 1 | S | 4 | S |
| Linezolid | 4 | S | 2 | S | 2 | S | 2 | S |
| Piperacillin/Tazobactam | 64/4 | R | 4/4 | S | >512/4 | R | 64/4 | R |
| Streptomycin | 8 | S | 2 | S | 64 | R | 64 | R |
| Tetracycline | 0.5 | S | 2 | S | 0.25 | S | 0.125 | S |
| Trimethoprim/Sulfamethoxazole | 0.063/1.2 | S | 0.125/2.4 | S | 8/152 | R | 8/152 | R |
| Vancomycin | 1 | S | 1 | S | 2 | S | 1 | S |

**MRSA (MT3302)**

| | MHB | | DMEM | | Serum | | Urine | |
|---|---|---|---|---|---|---|---|---|
| Antibiotic | MIC | Interpretation | MIC | Interpretation | MIC | Interpretation | MIC | Interpretation |
| Ampicillin | 256 | R | 32 | R | >512 | R | 64 | R |
| Azithromycin | 256 | R | 8 | R | 256 | R | 8 | R |
| Ceftriaxone | 64 | R | 8 | S | 128 | R | 16 | I |
| Cephalexin | 128 | R | 8 | S | 128 | R | 8 | S |
| Ciprofloxacin | 0.5 | S | 0.5 | S | 0.5 | S | 0.5 | S |
| Daptomycin | 1 | S | 0.125 | S | 4 | R | 0.5 | S |
| Ertapenem | 4 | I | 4 | I | 4 | I | 2 | S |
| Imipenem | 0.125 | S | 0.063 | S | 0.25 | S | 0.031 | S |
| Linezolid | 2 | S | 1 | S | 2 | S | 2 | S |
| Piperacillin/Tazobactam | 64/4 | R | 4/4 | S | 512/4 | R | 16/4 | R |
| Streptomycin | 8 | S | 2 | S | 32 | R | 1 | S |
| Tetracycline | 0.5 | S | 2 | S | 0.25 | S | 0.5 | S |
| Trimethoprim/Sulfamethoxazole | 0.063/1.2 | S | 0.125/2.4 | S | 8/152 | R | >32/608 | R |
| Vancomycin | 1 | S | 1 | S | 2 | S | 1 | S |

**MSSA (Newman)**

| | MHB | | DMEM | | Serum | | Urine | |
|---|---|---|---|---|---|---|---|---|
| Antibiotic | MIC | Interpretation | MIC | Interpretation | MIC | Interpretation | MIC | Interpretation |
| Ampicillin | >512 | R | 256 | R | >512 | R | 128 | R |
| Azithromycin | 1 | S | 0.063 | S | 2 | S | 64 | R |
| Ceftriaxone | 4 | S | 4 | S | 8 | S | 2 | S |
| Cephalexin | 32 | R | 4 | S | 1 | S | 2 | S |
| Ciprofloxacin | 0.125 | S | 0.25 | S | 0.25 | S | 1 | S |
| Daptomycin | 1 | S | 0.5 | S | 4 | R | 0.5 | S |
| Ertapenem | 0.5 | S | 1 | I | 0.125 | S | 0.25 | S |
| Imipenem | 0.016 | S | 0.031 | S | 0.008 | S | 0.016 | S |
| Linezolid | 4 | S | 2 | S | 2 | S | 2 | S |
| Piperacillin/Tazobactam | 2/4 | S | 2/4 | S | 4/4 | S | 1/4 | S |
| Streptomycin | 8 | S | 2 | S | 64 | R | 64 | R |
| Tetracycline | 0.5 | S | 2 | S | 0.25 | S | 0.125 | S |
| Trimethoprim/Sulfamethoxazole | 1/19 | S | 2/38 | S | 8/152 | R | 4/76 | R |
| Vancomycin | 1 | S | 1 | S | 2 | S | 1 | S |

***S. pneumoniae* (D39)**

| | MHB | | DMEM | | Serum | | Urine | |
|---|---|---|---|---|---|---|---|---|
| Antibiotic | MIC | Interpretation | MIC | Interpretation | MIC | Interpretation | MIC | Interpretation |
| Ampicillin | 0.016 | S | 0.016 | S | 0.016 | S | 0.016 | S |
| Azithromycin | 0.063 | S | <0.001 | S | 0.25 | S | 0.004 | S |
| Ceftriaxone | 0.008 | S | 0.016 | S | 0.25 | S | 0.016 | S |
| Cephalexin | 0.25 | S | 0.125 | S | 4 | I | 0.5 | S |
| Ciprofloxacin | 0.5 | I | 0.5 | I | 0.5 | I | 0.5 | I |
| Daptomycin | 0.25 | S | 0.031 | S | 4 | R | 1 | S |
| Ertapenem | 0.016 | S | 0.0625 | S | 0.016 | S | 0.016 | S |
| Imipenem | 0.004 | S | 0.002 | S | 0.002 | S | 0.001 | S |
| Linezolid | 0.5 | S | 0.5 | S | 1 | S | 0.5 | S |
| Piperacillin/Tazobactam | <0.001/4 | NA | <0.001/4 | NA | 0.001/4 | NA | 0.016/4 | NA |
| Streptomycin | 16 | NA | 2 | NA | 32 | NA | 8 | NA |
| Tetracycline | 0.125 | S | 0.25 | S | 0.125 | S | 0.25 | S |
| Trimethoprim/Sulfamethoxazole | 0.031/0.6 | S | 0.063/1.2 | S | 1/19 | I | 0.25/4.8 | S |
| Vancomycin | 0.25 | S | 0.25 | S | 0.5 | S | 0.25 | S |

***S. pneumoniae* (Daw 25)**

| | MHB | | DMEM | | Serum | | Urine | |
|---|---|---|---|---|---|---|---|---|
| Antibiotic | MIC | Interpretation | MIC | Interpretation | MIC | Interpretation | MIC | Interpretation |
| Ampicillin | 0.016 | S | 0.063 | S | 0.016 | S | 0.008 | S |
| Azithromycin | 0.125 | S | 0.008 | S | 0.5 | S | 0.031 | S |
| Ceftriaxone | 0.016 | S | 0.016 | S | 0.125 | S | 0.008 | S |
| Cephalexin | 2 | S | 4 | I | 2 | S | 0.25 | S |
| Ciprofloxacin | 0.5 | I | 0.5 | I | 1 | I | 0.063 | S |
| Daptomycin | 0.25 | S | 0.063 | S | 4 | R | 1 | S |
| Ertapenem | 0.008 | S | 0.125 | S | 0.016 | S | 0.016 | S |
| Imipenem | 0.008 | S | 0.031 | S | <0.001 | S | 0.002 | S |
| Linezolid | 0.5 | S | 1 | S | 1 | S | 0.25 | S |
| Piperacillin/Tazobactam | 0.004/4 | NA | 0.031/4 | NA | 0.008/4 | NA | 0.004/4 | NA |
| Streptomycin | 32 | NA | 4 | NA | 32 | NA | 16 | NA |
| Tetracycline | 0.125 | S | 0.5 | S | 0.125 | S | 0.063 | S |
| Trimethoprim/Sulfamethoxazole | 0.125/2.4 | S | 0.125/2.4 | S | 1/19 | I | 0.5/9.5 | S |
| Vancomycin | 0.25 | S | 0.5 | S | 0.5 | S | 0.25 | S |

**Clinical Breakpoints**

| Antibiotic | *Staphylococcus* spp. | *Enterococcus* spp. | *S. pneumoniae* |
|---|---|---|---|
| Ampicillin | S ≤ 0.25, R ≥ 0.5[1] | S ≤ 8, R ≥ 16 | S ≤ 0.5, R > 2[2] |
| Azithromycin | S ≤ 2, I = 4, R ≥ 8 | S ≤ 2, I = 4, R ≥ 8[3] | S ≤ 0.5, I = 1, R ≥ 2 |
| Ceftriaxone | S ≤ 8, I = 16-32, R ≥ 64[1] | Intrinsic Resistance | S ≤ 1, I = 2, R ≥ 4 |
| Cephalexin | S ≤ 8, I = 16, R ≥ 32[4] | Intrinsic Resistance | S ≤ 2, I = 4, R ≥ 8[5] |
| Ciprofloxacin | S ≤ 1, I = 2, R ≥ 4 | S ≤ 1, I = 2, R ≥ 4 | S ≤ 0.125, R > 2[6] |
| Daptomycin | S ≤ 1, NS ≥ 2 | S ≤ 4, NS ≥ 8 | R ≥ 2[7] |
| Ertapenem | S ≤ 2, I = 4, R ≥ 8[1] | S ≤ 0.5, R > 0.5[8] | S ≤ 1, I = 2, R ≥ 4 |
| Imipenem | S ≤ 4, I = 8, R ≥ 16[1] | S ≤ 4, R ≥ 8[8] | S ≤ 0.12, I = 0.25-0.5, R ≥ 1 |
| Linezolid | S ≤ 4, R ≥ 8 | S ≤ 2, I = 4, R ≥ 8 | R ≤ 2 |
| Piperacillin/Tazobactam | S ≤ 8/4, R ≥ 16/4[1] | S ≤ 16, R ≥ 32[9] | Not Available |
| Streptomycin | S ≤ 8, I = 16, R ≥ 32[10] | Intrinsic Resistance | Not Available |
| Tetracycline | S ≤ 4, I = 8, R ≥ 16 | S ≤ 4, I = 8, R ≥ 16 | S ≤ 1, I = 2, R ≥ 4 |
| Trimethoprim/Sulfamethoxazole | S ≤ 2/38, R ≥ 4/76 | Intrinsic Resistance | S ≤ 0.5/9.5, 1 = 1/19-2/38 R ≥ 4/76 |
| Vancomycin | S ≤ 2, I = 4-8, R ≥ 16 | S ≤ 4, I = 8-16, R ≥ 32 | ≤ 1 |

MICs and susceptibility designations were determined by broth microdilution in bacteriologic medium (MHB), mammalian cell culture medium (DMEM) and pooled human donor sera and urine (see Methods). MIC values were derived from the consensus of $\geq$ 6 independent determinations. Unless otherwise indicated,[1-10] all clinical breakpoints are referenced from CLSI, 2014.[11] *Staphylococcus* spp. breakpoints were used to interpret *S. aureus* MIC values; and *Enterococcus* spp. breakpoints used for *Enterococcus faecium.* *Indicates intrinsic resistance. Altered susceptibility designations are outlined in black boxes. S = susceptible; I = intermediate; R = resistant; NS = non-susceptible.

**Table S2. Gram-negative MICs and susceptibility designations from testing in bacteriologic medium, cell culture medium, and human sera or urine.** Related to Figure 1.

***A. baumannii*** **(19606)**

| | MHB | | DMEM | | Serum | | Urine | |
|---|---|---|---|---|---|---|---|---|
| Antibiotic | MIC | Interpretation | MIC | Interpretation | MIC | Interpretation | MIC | Interpretation |
| Ampicillin* | 256 | R | 64 | R | 64 | R | 64 | R |
| Azithromycin* | 16 | R | 8 | R | 32 | R | 512 | R |
| Ceftriaxone | 32 | I | 16 | I | 32 | I | 16 | I |
| Cephalexin* | >512 | R | >512 | R | >512 | R | 512 | R |
| Ciprofloxacin | 0.5 | S | 1 | S | 1 | S | 4 | R |
| Colistin Sulfate | 0.5 | S | 4 | R | 0.125 | S | 2 | S |
| Ertapenem* | 4 | R | 8 | R | 1 | R | 2 | R |
| Imipenem | 0.25 | S | 0.25 | S | 0.031 | S | 0.25 | S |
| Piperacillin/Tazobactam | ≤0.001/4 | S | ≤0.001/4 | S | ≤0.001/4 | S | 16/4 | S |
| Streptomycin | 512 | R | 64 | R | 128 | R | 512 | R |
| Tetracycline | 2 | S | 32 | R | 0.5 | S | 0.5 | S |
| Trimethoprim/Sulfamethoxazole | 16/304 | R | 16/304 | R | 4/76 | R | 16/304 | R |

***E. cloacae*** **(13047)**

| | MHB | | DMEM | | Serum | | Urine | |
|---|---|---|---|---|---|---|---|---|
| Antibiotic | MIC | Interpretation | MIC | Interpretation | MIC | Interpretation | MIC | Interpretation |
| Ampicillin | >512 | R | >512 | R | >512 | R | 512 | R |
| Azithromycin | 16 | S | 2 | S | 16 | S | >512 | R |
| Ceftriaxone | 4 | R | 0.25 | S | 256 | R | 16 | R |
| Cephalexin | >512 | R | >512 | R | >512 | R | >512 | R |
| Ciprofloxacin | 0.016 | S | 0.008 | S | 0.125 | S | 0.5 | S |
| Colistin Sulfate | 32 | R | >512 | R | 64 | R | >512 | R |
| Ertapenem | 0.25 | S | 4 | R | 4 | R | 0.125 | S |
| Imipenem | 1 | S | 2 | I | 2 | I | 1 | S |
| Piperacillin/Tazobactam | 16/4 | S | 4/4 | S | 128/4 | R | >512/4 | R |
| Streptomycin | >512 | R | >512 | R | >512 | R | >512 | R |
| Tetracycline | 2 | S | 8 | I | 2 | S | 2 | S |
| Trimethoprim/Sulfamethoxazole | 1/19 | S | 0.25/4.8 | S | 4/76 | R | 32/608 | R |

***E. coli*** **(25922)**

| | MHB | | DMEM | | Serum | | Urine | |
|---|---|---|---|---|---|---|---|---|
| Antibiotic | MIC | Interpretation | MIC | Interpretation | MIC | Interpretation | MIC | Interpretation |
| Ampicillin | 4 | S | 4 | S | 2 | S | 2 | S |
| Azithromycin | 4 | S | 1 | S | 4 | S | 128 | R |
| Ceftriaxone | 0.063 | S | 0.016 | S | 0.5 | S | 0.125 | S |
| Cephalexin | 8 | S | 8 | S | 8 | S | 16 | S |
| Ciprofloxacin | 0.004 | S | 0.004 | S | 0.016 | S | 0.063 | S |
| Colistin Sulfate | 0.25 | S | 0.5 | S | 0.125 | S | 0.125 | S |
| Ertapenem | 0.016 | S | 0.031 | S | 0.031 | S | 0.016 | S |
| Imipenem | 0.25 | S | 1 | S | 0.25 | S | 0.125 | S |
| Piperacillin/Tazobactam | 2/4 | S | 2/4 | S | 2/4 | S | 4/4 | S |
| Streptomycin | 8 | S | 1 | S | 4 | S | 32 | R |
| Tetracycline | 1 | S | 8 | I | 1 | S | 0.5 | S |
| Trimethoprim/Sulfamethoxazole | 0.063/1.2 | S | 0.063/1.2 | S | 0.5/9.5 | S | 4/76 | R |

***K. pneumoniae*** **(13883)**

| | MHB | | DMEM | | Serum | | Urine | |
|---|---|---|---|---|---|---|---|---|
| Antibiotic | MIC | Interpretation | MIC | Interpretation | MIC | Interpretation | MIC | Interpretation |
| Ampicillin | 256 | R | 512 | R | 128 | R | 512 | R |
| Azithromycin | 4 | S | 2 | S | 4 | S | 256 | R |
| Ceftriaxone | 0.063 | S | 0.125 | S | 1 | S | 0.125 | S |
| Cephalexin | 8 | S | 16 | S | 8 | S | 16 | S |
| Ciprofloxacin | 0.031 | S | 0.016 | S | 0.063 | S | 0.125 | S |
| Colistin Sulfate | 0.25 | S | 16 | R | 0.063 | S | 1 | S |
| Ertapenem | 0.016 | S | 0.063 | S | 0.031 | S | 0.031 | S |
| Imipenem | 0.5 | S | 2 | I | 2 | I | 0.5 | S |
| Piperacillin/Tazobactam | 2/4 | S | 4/4 | S | 1/4 | S | 4/4 | S |
| Streptomycin | 2 | S | 1 | S | 2 | S | 16 | I |
| Tetracycline | 1 | S | 16 | R | 1 | S | 1 | S |
| Trimethoprim/Sulfamethoxazole | 0.125/2.4 | S | 0.031/0.6 | S | 0.25/4.8 | S | 2/38 | S |

***K. pneumoniae*** **(MT3325)**

| | MHB | | DMEM | | Serum | | Urine | |
|---|---|---|---|---|---|---|---|---|
| Antibiotic | MIC | Interpretation | MIC | Interpretation | MIC | Interpretation | MIC | Interpretation |
| Ampicillin | >512 | R | >512 | R | >512 | R | >512 | R |
| Azithromycin | 128 | R | 128 | R | 8 | S | >512 | R |
| Ceftriaxone | >512 | R | 64 | R | >512 | R | >512 | R |
| Cephalexin | >512 | R | >512 | R | >512 | R | >512 | R |
| Ciprofloxacin | 128 | R | 64 | R | 256 | R | >512 | R |
| Colistin Sulfate | 0.125 | S | 1 | S | 0.063 | S | 0.5 | S |
| Ertapenem | 256 | R | 64 | R | 32 | R | 64 | R |
| Imipenem | 32 | R | 16 | R | 16 | R | 8 | R |
| Piperacillin/Tazobactam | >512/4 | R | 512/4 | R | >512/4 | R | >512/4 | R |
| Streptomycin | 128 | R | 32 | R | 128 | R | 512 | R |
| Tetracycline | 4 | S | 16 | R | 2 | S | 2 | S |
| Trimethoprim/Sulfamethoxazole | >32/608 | R | >32/608 | R | >32/608 | R | >32/608 | R |

***P. aeruginosa*** **(10145)**

| | MHB | | DMEM | | Serum | | Urine | |
|---|---|---|---|---|---|---|---|---|
| Antibiotic | MIC | Interpretation | MIC | Interpretation | MIC | Interpretation | MIC | Interpretation |
| Ampicillin* | 128 | R | 256 | R | 16 | R | >512 | R |
| Azithromycin* | 64 | R | 32 | R | 128 | R | 512 | R |
| Ceftriaxone* | 8 | R | 8 | R | 16 | R | 8 | R |
| Cephalexin* | >512 | R | >512 | R | >512 | R | >512 | R |
| Ciprofloxacin | 0.125 | S | 0.25 | S | 0.125 | S | 0.5 | S |
| Colistin Sulfate | 0.5 | S | 8 | R | 0.5 | S | 0.5 | S |
| Ertapenem* | 4 | R | 4 | R | 4 | R | 4 | R |
| Imipenem | 0.5 | S | 0.5 | S | 2 | S | 1 | S |
| Piperacillin/Tazobactam | 4/4 | S | 4/4 | S | 0.5/4 | S | 4/4 | S |
| Streptomycin | 32 | R | 32 | R | 32 | R | 256 | R |
| Tetracycline* | 64 | R | >512 | R | 32 | R | 32 | R |
| Trimethoprim/Sulfamethoxazole* | 32/608 | R | 4/76 | R | 4/76 | R | 8/152 | R |

***S.*** **Typhimurium (14028)**

| | MHB | | DMEM | | Serum | | Urine | |
|---|---|---|---|---|---|---|---|---|
| Antibiotic | MIC | Interpretation | MIC | Interpretation | MIC | Interpretation | MIC | Interpretation |
| Ampicillin | 1 | S | 1 | S | 0.25 | S | 0.5 | S |
| Azithromycin | 4 | S | 1 | S | 2 | S | 256 | R |
| Ceftriaxone | 0.063 | S | 0.031 | S | 0.25 | S | 0.063 | S |
| Cephalexin | 4 | S | 8 | S | 4 | S | 8 | S |
| Ciprofloxacin | 0.016 | S | 0.008 | S | 0.031 | S | 0.125 | I |
| Colistin Sulfate | 0.25 | S | 2 | S | 0.125 | S | 0.25 | S |
| Ertapenem | 0.008 | S | 0.031 | S | 0.016 | S | 0.016 | S |
| Imipenem | 0.125 | S | 0.5 | S | 0.125 | S | 0.125 | S |
| Piperacillin/Tazobactam | 2/4 | S | 1/4 | S | 0.5/4 | S | 2/4 | S |
| Streptomycin | 16 | I | 4 | S | 128 | R | 128 | R |
| Tetracycline | 1 | S | 8 | I | 0.5 | S | 1 | S |
| Trimethoprim/Sulfamethoxazole | 0.063/1.2 | S | 0.063/1.2 | S | 0.125/2.4 | S | 16/304 | R |

**Clinical Breakpoints**

| Antibiotic | Enterobacterales | *P. aeruginosa* | *Acinetobacter* spp. |
|---|---|---|---|
| Ampicillin | S ≤ 8, I = 16, R ≥ 32 | Intrinsic Resistance | Intrinsic Resistance |
| Azithromycin | S ≤ 16, R ≥ 32[12] | Intrinsic Resistance | Intrinsic Resistance |
| Ceftriaxone | S ≤ 1, I = 2, R ≥ 4 | Intrinsic Resistance | S ≤ 8, I = 16-32, R ≥ 64 |
| Cephalexin | S ≤ 16, R > 16[12] | Intrinsic Resistance | Intrinsic Resistance |
| Ciprofloxacin** | S ≤ 1, I = 2, R ≥ 4 | S ≤ 1, I = 2, R ≥ 4 | S ≤ 1, I = 2, R ≥ 4 |
| Colistin Sulfate | S ≤ 2, R ≥ 4[12] | S ≤ 2, I = 4, R ≥ 8 | S ≤ 2, R ≥ 4 |
| Ertapenem | S ≤ 0.5, I = 1, R ≥ 2 | Intrinsic Resistance | Intrinsic Resistance |
| Imipenem | S ≤ 1, I = 2, R ≥ 4 | S ≤ 2, I = 4, R ≥ 8 | S ≤ 2, I = 4, R ≥ 8 |
| Piperacillin/Tazobactam | S ≤ 16/4, I = 32/4-64/4, R ≥ 128/4 | S ≤ 16/4, I = 32/4-64/4, R ≥ 128/4 | S ≤ 16/4, I = 32/4-64/4, R ≥ 128/4 |
| Streptomycin | S ≤ 8, I = 16, R ≥ 32[10] | S ≤ 8, R > 16[13] | S ≤ 8, R > 16[13] |
| Tetracycline | S ≤ 4, I = 8, R ≥ 16 | Intrinsic Resistance | S ≤ 4, I = 8, R ≥ 16 |
| Trimethoprim/Sulfamethoxazole | S ≤ 2/38, R ≥ 4/76 | Intrinsic Resistance | S ≤ 2/38, R ≥ 4/76 |

MICs and susceptibility designations were determined by broth microdilution in bacteriologic medium (MHB), mammalian cell culture medium (DMEM) and pooled human donor sera and urine (see Methods). MIC values were derived from the consensus of $\geq$ 6 independent determinations. Unless otherwise indicated,[1-4] all clinical breakpoints are referenced from CLSI, 2014.[11] Enterobacterales breakpoints were used to interpret *Salmonella*, *Escherichia*, *Klebsiella*, and *Enterobacter* MIC values, with azithromycin breakpoints referenced from CLSI, 2022, EUCAST, 2023, Gomes et al.[12,14-16]; *Acinetobacter* spp. breakpoints were applied to *A. baumannii*. *Indicates intrinsic resistance. *******Salmonella* ciprofloxacin clinical breakpoints: S ≤ 0.06, I = 0.125-0.5, R ≥ 1. Altered susceptibility designations are outlined in black boxes. S = susceptible; I = intermediate; R = resistant.

## Supplementary Figure

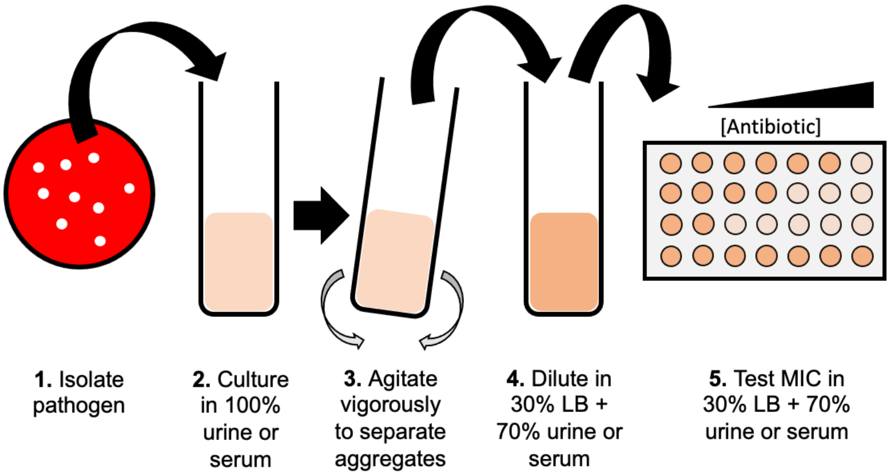


**Figure S1. Overview of AST protocol for testing in human serum and urine.** Related to STAR Methods. 1) Bacterial pathogens are isolated; 2) grown in 100% serum or urine; 3) agitated to separate cell aggregates; 4) diluted into supplemented human fluids (30% LB + 70% urine or serum); and 5) MIC testing is performed in supplemented human fluids in microtiter plates.

## References Cited


[S1] Clinical and Laboratory Standards Institute. Methods for dilution antimicrobial susceptibility tests for bacteria that grow aerobically; Approved standard-ninth edition. 2012.

[S2] European Committee on Antimicrobial Susceptibility Testing. Breakpoint tables for interpretation of MICs and zone diameters. Version 11.02021. http://www.eucast.org (accessed August 30, 2021).

[S3] Fass R. Erythromycin, clarithromycin, and azithromycin: use of frequency distribution curves, scattergrams, and regression analyses to compare in vitro activities and describe cross-resistance. Antimicrob Agents Chemother 1993; **37**(10): 2080-6.

[S4] Teva Parmaceuticals. Cephalexin Capsules USP [Product Insert]. Sellersville, PA, 2012.

[S5] Clinical and Laboratory Standards Institute. Performance standards for antimicrobial disk and dilution susceptibility tests for bacteria isolated from animals. 5th ed. CLSI supplement VET01S. 2020.

[S6] Schurek K, Adam H, Hoban D, Zhanel G. Call for the international adoption of microbiological breakpoints for fluoroquinolones and Streptococcus pneumoniae. Intl J Antimicrob Agents 2006; **28**(3): 266-9.

[S7] Wise R, Andrews J, Ashby J. Activity of daptomycin against Gram-positive pathogens: a comparison with other agents and the determination of a tentative breakpoint. J Antimicrob Chemother 2001; **48**(4): 563-7.

[S8] European Committee on Antimicrobial Susceptibility Testing. Breakpoint tables for interpretation of MICs and zone diameters. Version 9.0. 2019. https://www.eucast.org/ast_of_bacteria/previous_versions_of_documents/ (accessed October 30, 2022).

[S9] Hällgren A, Abednazari H, Ekdahl C, et al. Antimicrobial susceptibility patterns of enterococci in intensive care units in Sweden evaluated by different MIC breakpoint systems. J Antimicrob Chemother 2001; **48**(1): 53-62.

[S10] Societe Francaise de Microbiologie. Comite de l'Antibiogramme de la Societe Francaise de Microbiologie (CASFM). 2012.

[S11] Clinical and Laboratory Standards Institute. Performance standards for antimicrobial resistance testing; twenty-fourth informational supplement, M100-S24, 2014.

[S12] European Committee on Antimicrobial Susceptibility Testing. Breakpoint tables for interpretation of MICs and zone diameters. Version 6.0. 2016.

[S13] Societe Francaise de Microbiologie. SFM Antibiogram Committee, Comite de l'Antibiogramme de la Societe Francaise de Microbiologie, report 2003. Itnl J Antimicrob Agents 2003; **21**: 364-91.

[S14] European Committee on Antimicrobial Susceptibility Testing. Clinical breakpoints- breakpoints and guidance, 2023. https://www.eucast.org/clinical_breakpoints (accessed February 8, 2023).

[S15] Clinical and Laboratory Standards Institute. Performance standards for antimicrobial susceptibility testing, M100, 32nd ed, 2022. (accessed February 8, 2023).

[S16] Gomes C, Ruiz-Roldán L, Mateu J, Ochoa T, Ruiz J. Azithromycin resistance levels and mechanisms in Escherichia coli. Sci Rep, 2019. https://doi.org/10.1038/s41598-019-42423-3 (accessed February 8, 2023).
